# International Predictors of Contract Cheating in Higher Education

**DOI:** 10.1007/s10805-022-09449-1

**Published:** 2022-04-08

**Authors:** R. Awdry, B. Ives

**Affiliations:** 1 Centre for Research in Assessment and Digital Learning, Deakin University, Melbourne, Australia; 2 Professional Specialized Studies Division, University of Nevada, Reno, USA

**Keywords:** Assignment outsourcing, Essay mills, Contract cheating, Cheating motivations, Peer cheating, Academic integrity

## Abstract

Prevalence of contract cheating and outsourcing through organised methods has received interest in research studies aiming to determine the most suitable strategies to reduce the problem. Few studies have presented an international approach or tested which variables could be correlated with contract cheating. As a result, strategies to reduce contract cheating may be founded on data from other countries, or demographics/situations which may not align to variables most strongly connected to engagement in outsourcing. This paper presents the results of a series of statistical analyses aimed at testing which variables were found to be predictors of students’ self-reported formal outsourcing behaviours. The data are derived from an international research study conducted in 22 languages, with higher education students (from Europe, the Americas and Australasia. Analyses found that country and discipline of study as well as the rate at which respondents n = 7806) believed other students to be cheating, were positively correlated to their cheating behaviours. Demographic variables did not show strong statistical significance to predicting contract cheating.

## Introduction

The use of essay mills in higher education appears to be increasing. An historical international average of 3.52% for contract cheating use amongst university students was reported by Newton (2018), following analysis of 65 studies totalling 54,514 respondents. He noted that since 2014, the percentage of students who admitted having paid someone to do their assignment increased to 15.7%. However, rates may have been affected by differing definitions of contract cheating. Curtis and Clare (2017) reported a rate of 3.5% of contract cheating in their research, whilst Bretag et al., (2018b), reported an overall self-reported engagement rate of 5.78% in their defined contract cheating behaviours. Awdry (2020), found self-reported outsourcing rates of 7.4% with formal outsourcing methods (contract cheating); and a 16.9% average for all outsourcing methods (paid or unpaid, and inclusive of companies, friends family or other students).

Whilst this indicates a minority of students are engaging in these behaviours, when coupled with a disturbing prevalence of essay mills and other types of sites offering help to students struggling with their assignments in highly organised business structures (Ellis et al., 2018), these numbers are concerning. Advertisements for cheating sites are ubiquitous in social media and search engines. Many companies have targeted marketing campaigns, quality and product guarantees, and promote their business as though it is merely a study aid, normalising engagement with their services (Amigud, 2019; Medway et al., 2018; Rowland et al., 2018). Students do not associate some sites with cheating at all (Harrison et al., 2020). Existing studies have demonstrated rates of outsourcing and usage of different sites, as well as self-reported motivations for why students engage in cheating in this manner, but less focus has been placed on which variables may predict contract cheating. We add to the literature by providing a statistical focus to the problem through analysis of different variables in order to determine which variables may be related to contract cheating.

## Literature Background

Commercial contract cheating sites have varying business models which offer work to students, for example: essay mills (usually offering pre-written work to students); peer sharing sites (users can download and upload material from the database); bespoke assignment sites (where items are made-to-order); bidding sites (which allow users to post requirements and accept the most suitable offer to their monetary/time and other requirements). Derived from Awdry's (2020), definition of assignment outsourcing, throughout this paper, all site types will be referred to as ‘formal outsourcing’.

Self-report studies conducted in different countries and disciplines have found some correlation internationally in variables associated with cheating. Motivational or situational variables that have been linked to cheating, whether specifically to outsourcing or more broadly to plagiarism, include: lack of time; fear of failure or pressure to achieve high grades; disengagement with learning/lack of motivation to learn; lack of understanding of requirements; university sanctions; culture of cheating or peer cheating influences (Bowers, 1964; Brimble & Stevenson-Clarke, 2005; Devlin & Gray, 2007; Genereux & McLeod, 1995; Gullifer & Tyson, 2010; Haines et al., 1986; McCabe & Bowers, 1994; Molnar & Kletke, 2012; Tomar, 2015; Whitley, 1998). Individual variables have commonly been associated with students who are male, have a low-grade point average (GPA), and are younger or studying the lower academic levels (Brimble, 2016; Chapman et al., 2004; McCabe & Bowers, 1994; McCabe & Trevino, 1997; Park, 2003; Underwood & Szabo, 2003; Whitley & Keith-Spiegel, 2001). Students self-reporting cheating have also more commonly reported witnessing or believing their peers to be cheating, and may therefore rationalise their engagement in cheating due to perceptions of normalisation (McCabe, 2005; McCabe & Trevino, 1993; Rettinger & Kramer, 2009; Rigby et al., 2015).

Less research has been done which specifically looks at the factors or variables associated with student engagement with contract cheating. However, congruent with research on cheating more generally, studies on contract cheating, essay mills and sharing behaviours have found that younger students, males and students studying in their second language may be more likely to outsource (Bretag et al., 2018a, b). Although Awdry and Ives (2020), found that when students outsourced from those they knew, this was more common amongst first language learners. Disengagement or dissatisfaction with the learning environment or assessment task, fear of failure, as well as cultures of cheating which may normalise contract cheating have also been seen to affect the rates at which students choose to outsource. Bretag et al., (2018a), and Lancaster and Clarke, (2016), found that some assessment types were associated with higher contract cheating.

Cheating has been seen to decrease as students moved up university levels, and that a lack of time, misunderstanding the topic or laziness contributed to outsourcing behaviours (Foltýnek & Králíková, 2018). Tremayne and Curtis (2020) found that personality traits could be correlated to cheating. Dante (pseudonym for Dave Tomar, essay writer) (2010) commented that students buying assignments from him were often lazy, had disengaged from the learning environment or had poor academic skills. Perceptions that there are lots of opportunities to cheat are also statistically significant contributors to outsourcing behaviours (Bretag et al., 2018a, b).

Very few studies have tested the statistical significance of situational or personal variables against a student’s engagement with contract cheating. Without knowing what factors can lead to cheating, interventions or strategies cannot be proposed to address these behaviours. Given that research into interventions to improve academic integrity is limited and often have poor inference quality (Ives & Nehrkorn, 2019), the foundations for developing effective interventions are essential. Descriptive data on contract cheating in the aforementioned research helps to understand the extent of the problem but does not inform educators and legislators which factors may predict students’ engagement in contract cheating, restricting the ability to implement proactive strategies informed by empirical evidence.

Additionally, only a handful of data are available to present a comparable picture of the prevalence, types, and motivations for formal outsourcing globally. Without an internationally comparable data set, effective responses may not address the specific types of outsourcing behaviours found in different countries. Rates of cheating reported from different countries vary, and university responses to the problem may be based on a prevalence rate of cheating substantially different to actual rates in their local context. This project was designed to gather an in-depth insight into these types of behaviours globally and statistically test variables for the effect they may have on engagement with outsourcing. While correlational and descriptive studies do not evidence causation, they allow an understanding of the association of certain variables (the independent variables tested) against the dependent variable (engagement in outsourcing). This knowledge will provide empirical evidence to educators when designing strategies targeted at those associations. It is hoped that this will allow institutions internationally to use relevant data for their context to consider suitable and useful strategies to reduce motivations towards the use of different methods of commercial assignment outsourcing sites.

The paper reports on outcomes from an international study which surveyed students internationally on their use and knowledge of, motivations for, and engagement with, different types of formal outsourcing methods. The data presented here will respond to the following research questions:

- R1—Are there personal or situational variables which have a correlation with usage of assignment outsourcing sites?
- R2—Do students who outsource through formal methods have higher rates of known or perceived cheating amongst their peer groups and the general student population?
- R3—Do the students who outsource their assignments from sites agree with more factors as to why it is acceptable to cheat?

Statistical significance testing of personal and situational variables to respond to the above questions are presented below. We acknowledge that statistical significance may not have such strengths in practical application, although after presenting the results, we provide a discussion to consider what implications the data may have for educators. We also discuss the limitations of the study and conclude with possible policy implications and practical actions that institutions could take. Although this study was undertaken prior to the COVID-19 pandemic, it may provide a helpful context regarding possible financial and study pressures which might be exacerbated for students.

## Methods

Primary objectives of the project were to understand the prevalence of different types of outsourcing globally, as well as the factors which may contribute to it, and students’ knowledge about the legality of essay mills and usage amongst peers (for a full background and descriptive statistics, see Awdry, 2020). We wanted to compare survey outcomes with prior research findings and published studies, allowing them to be interpreted and applied across a range of educational contexts and languages. In the survey, we defined different types of outsourcing for participants, which included formal and informal outsourcing methods, for this paper we focus solely on formal outsourcing (for the analysis of informal methods, please refer to Awdry and Ives, 2020). By providing a response matrix, we asked respondents to select the mode through which they had obtained the outsourced assignment, whether this was for free, in exchange for something else, or for money. This context was considered necessary to more fully understand how students obtain assignments.

Data were gathered through a self-report survey designed in four sections, including qualitative and quantitative questions, asking students to provide information on their study behaviours, their knowledge of others’ study behaviours, outcomes for cheating, and demographic information. No identifying information was requested, although respondents could voluntarily provide their contact details at the end of the survey. Please refer to Awdry et al. (2020) to view the survey.

To investigate content validity for the instrument, the English survey was piloted with students and prominent researchers in higher education. These experts in the field provided feedback on how well the content of the instrument aligned with the constructs we intended to measure. In general, this feedback was supportive of the content validity of the instrument. We also piloted the instrument with post-secondary students to get their feedback about how understandable the survey items were in terms of both format and language. Again, the feedback we received was generally positive. The final English language survey was translated into twenty-one other languages. To mitigate for possible misunderstandings and content validity limitations, following recommendations by Junger-Tas and Marshall (1999), we carried out pilots in all languages to establish understanding, particularly in the local context. These were completed by persons with fluency or native language skills and were subsequently piloted by a minimum of two persons per language. Each survey was released for thirteen weeks between 2017 and 2018 through convenience and snowballing sampling methods due to the complex nature of disseminating a survey in multiple countries simultaneously.

Our approach to data analysis was first to identify an outcome (dependent) variable and which predictor (independent) variables would correspond to our research questions, based on our specific survey items. For data analysis, the term ‘Site’ is used to categorise the four types of formal outsourcing methods we explored. Second, bivariate analyses were used to determine which individual variables accounted for a significant amount of the total variance for the outcome variable using Pearson correlations or one-way analysis of variance (ANOVA), depending on whether the predictors were treated as continuous or categorical, respectively. Given the large sample size for this study, we had ample statistical power to find statistical significance for very small effects. Third, we recognized that the bivariate analyses did not account for covariance between predictors. For this reason, we ran a regression using all the predictors that individually accounted for at least one percent of the outcome variable.

While conventional significance testing is useful in determining the probability of Type 1 errors, these tests have some limitations. Because these tests are sensitive to sample size, they can identify effects as statistically significant, but they do not reflect the size of those effects. In other words, the same mean difference between groups, or correlation coefficient may not be statistically significant in one study, but significant in another, simply because the latter study had a larger sample size. Other things being equal (e.g., variance) the larger the sample size, the more statistical power is available to find statistical significance even in very small effects that may not justify practical attention (Ives, 2003).

For this reason, a substantial amount of variance was defined using Cohen's (1988) guidelines. Correlations of at least 0.10 (1% of the total variance) and standardized mean differences of at least 0.20 (also 1% of the total variance) were considered substantial. Cohen suggested these criteria as minimums for small effects. Finally, substantial predictors were entered into a general linear model to determine to what extent predictors contribute independent variance to the outcome variable.

As not all questions were mandatory, we experienced drop-out of approximately 33%; numbers of participants varied across sections and items. An initial 10,495 students accessed the survey. We provide response numbers for specific items where useful in the presented results. For the survey items considered for this analysis we received approximately 7800 responses (74% response rate of those accessing the survey).

## Results

### Outcome Variable

Several items on the instrument asked participants how many times (Never, 1–2, 3–4, 5 + or All assignments) they used sites for their academic work. The question was asked about four types of outsourcing (essay mills, peer-sharing, essay bidding and contract essay sites), and by which mode participants had obtained work: with money, for free, or by exchanging a document or information.

**Fig. 1:**
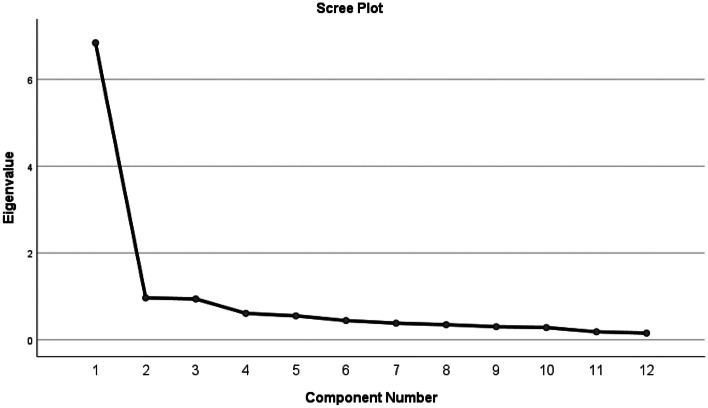
Scree Plot for Factor Analysis of Responses to Using Sites to Obtain Work

We used exploratory factor analysis (see Fig. 1) to explore the dimensionality of these twelve times, applying principal components analysis, and varimax rotation. The purpose of exploratory factor analysis is to investigate the underlying structure of responses to multiple items to determine the construct validity of that structure (e.g. Canivez et al., 2021). The scree plot for these items suggests a single factor with an eigen value of 6.84, accounting for 57.00 percent of the total variance. No other factors had eigen values above one. Because a single factor was identified, the solution was not rotated.

Treating all 12 items as a single factor (see Table 1), the internal consistency reliability was strong (Cronbach’s alpha = 0.922). Considering these results, we conducted analyses based on a single outcome variable: **SiteUse** (sum of responses for the 12 items involving interactions with sites). The results for the factor analysis and Cronbach’s alpha demonstrate strong construct validity for the SiteUse outcome variable.

**Table 1:** Item Loadings for a Single Outcome Factor

Component Matrix	
	Component 1
BuyMill	0.770
BuyPeer	0.691
BuyBid	0.832
BuyCont	0.763
ExchMill	0.774
ExchPeer	0.715
ExchBid	0.844
ExchCont	0.808
FreeMill	0.598
FreePeer	0.595
FreeBid	0.813
FreeCont	0.805

### Continuous Predictor Variables

Various items on the survey asked participants about their behaviours or the behaviours of others, which we wanted to test as our predictor variables. These included:

- Reason—the sum of coded scores for a mandatory item that asked participants why they chose to come to university. Participants were given seven specific options, in addition to open text for ‘Other’. More than one could be selected. Three options were coded as positive one (+ 1) because they related to internal motivation based on the value of learning, and four coded as negative one (-1) because they did not reflect this internal value of learning. The sum was treated as a continuous variable reflecting a range from intrinsic to extrinsic motivations for university enrolment.
- Tutor—a ranked variable based on responses to: ‘Do you think that any of your tutors know you by name?’ The item was mandatory with the options: None, Some, and All, coded 0, 1, and 2, respectively.
- UseRate—asked participants, ‘What proportion of students do you think uses these sites?’ (mandatory)
- StudyLevel—a ranked variable based on responses given to an optional item, asking if they were: undergraduate/bachelor, postgraduate/masters, or research/PhD.
- Age—based on age in years (optional).
- CheatingAcceptable—a variable from one mandatory item asking respondents under what conditions cheating was acceptable. Six different situations were offered, along with an option of ‘Never’. Each ‘Yes’ response created the CheatingAcceptable variable.

### Categorical Predictor Variables

- OthersSiteUse – a dichotomous variable (mandatory), ‘Are you aware of any of your friends or peers having used these sites (either essay mills, exchange sites, peer sharing sites or assignment bidding sites)?’
- Country – based on responses asking each participant where they were studying (optional). Each country was assigned a unique numerical code. Participants reported studying in 54 different countries.
- Discipline – optional item asking which category best described respondent’s field of study. Responses were based on the Australian Standard Classification of Education discipline codes, which refer to UNESCO codes.
- Gender – a single optional survey item with three options: male, female, and indeterminate, coded 1, 2, and 3, respectively.
- Language – a single mandatory item with two options: Are you studying in your first or second language? Coded 0 for first, and 1, for second.

### Correlations with Continuous Predictor Variables

To respond to our research questions wanting to know whether any personal or situational variables correlated with site usage, as well as whether those students who outsource respond positively to situations when cheating may be considered acceptable, we ran two-tailed Pearson correlations to determine which of the continuous predictor variables had at least a small effect (*r* = 0.10, *r*^*2*^ = 0.01), based on Cohen’s guidelines, with SiteUse. The results as shown in Table 2. The proportion of variance explained is reported only if it is over one percent.

**Table 2:** Correlations (Proportion of Variance) to SiteUse

Variable		SiteUse
Age	Pearson Correlation	-0.001
	Sig. (2-tailed)	0.903
	N	6883
StudyLevel	Pearson Correlation	0.053
	Sig. (2-tailed)	0.000
	N	6999
UseRate	Pearson Correlation	0.138 (0.019)
	Sig. (2-tailed)	0.000
	N	7316
Tutor	Pearson Correlation	0.026
	Sig. (2-tailed)	0.022
	N	7678
Reason	Pearson Correlation	-0.039
	Sig. (2-tailed)	0.001
	N	7665
CheatingAcceptable	Pearson Correlation	0.049
	Sig. (2-tailed)	0.008
	N	2949

While most of these correlations are statistically significant based on a conventional alpha level of 0.05, the table demonstrates that UseRate is the only continuous predictor that explains more than one percent of the total variance for SiteUse. In answer to our second research question: ‘Do students who outsource through formal methods have higher rates of known or perceived cheating amongst their peer groups and the general student population?’, we found that yes, students who believed that other students used sites at a higher rate were more likely to use these sites than students who believed that other students used sites at a lower rate. This correlation accounted for about 1.9%, which is a small effect. These results also show that the sample size was large enough to detect effects accounting for less than one percent of the variance.

The CheatingAcceptable variable represents situations in which participants might find the use of essay mills acceptable. Because these situations may be somewhat independent, we ran additional correlations between responses for each situation and the use of essay mill sites. Results are shown in Table 3. All of the correlations were significant at *p* < 0.0005 (*n* = 7806). In considering when cheating may be acceptable, the following reasons had the strongest correlations:, ‘If you don’t understand the topic’, ‘If the module/unit/subject is compulsory and you don’t want to study it’, and ‘If you don’t see the purpose of the task you are asked to complete’. However, none of them accounted for more than one percent of the variance. Therefore, in responding to the third research question: ‘Do the students who outsource their assignments from sites agree with more factors as to why it is acceptable to cheat?’, we found that there were significant correlations but they did not meet the required level to determine that this is a predictor of formal outsourcing.

**Table 3:** Correlations between Acceptable Situations (for cheating) and Essay Mill Use

Situation	Correlation
If you don't understand the topic	0.072
If you ran out of time due to other pressures	0.039
If you don't get enough time or support from your tutors	0.041
If you have too many assignments due at the same time from different classes	0.041
If you don't see the purpose of the task you are asked to complete	0.060
If the module/unit/subject is compulsory and you don't want to study it	0.061

### Mean Differences across Categorical Predictor Variables

We ran one-way analyses of variance (ANOVA) to determine which categorical predictors had at least a small effect, based on Cohen’s guidelines. These categorical analyses tested R1 and R2: whether students with knowledge of others’ cheating correlated with self-reported cheating behaviours; whether international patterns were seen; and to test other (categorical) personal/situational variables compared to site usage. In each case, we reported results for the Welch test when the test of homogeneity of variance failed. Pairwise Tukey tests were used to determine differences between pairs of categories, when a variable included more than two groups.

For OthersSiteUse, the one-way ANOVA test was statistically significant (*p* < 0.0005) for SiteUse. Students who reported being aware of others using sites were significantly more likely to outsource their own work. The effect size was small (*d* = 0.248). As with the UseRate that students believed the general student population were using formal outsourcing, the knowledge of peers using essay mills also affirmed the second research questions, that students who outsource through formal methods know more peers who cheat.

The one-way ANOVA test was statistically significant (*p* < 0.0005) for Country. Because the Statistical Package for Social Sciences (SPSS) will not run post hoc analyses on more than 50 groups, we eliminated countries with fewer than 10 participants from the post hoc analyses. We discussed here only the first 15 countries (concerning the highest response numbers), however, statistics for all countries with more than 10 participants can be found in the appendices. The following data (Table 4) report means and standard deviations (SD) of the SiteUse scores for each country. Countries are ordered from the lowest to the highest mean use of outsourcing sites. Note that for countries with fewer participants, the estimates for the means will be less reliable, and for that reason, those means are less likely to be significantly different from other means.

**Table 4:** Means and Standard Deviations of SiteUse Scores by Country

Country	N	Mean	SD
Sweden	1142	0.0193	0.26075
United Kingdom	63	0.0317	0.25198
Australia	1340	0.0358	0.33052
Italy	237	0.1266	0.82391
Czech Republic	575	0.1461	0.69056
Chile	1048	0.1498	0.62538
Hungary	208	0.1538	0.91966
New Zealand	76	0.1711	1.17062
United States	99	0.2424	0.90453
Republic of Serbia	1211	0.2733	1.85609
Romania	359	0.4401	1.35826
Montenegro	64	0.5469	1.79885
Turkey	166	0.5783	2.28365
Slovakia	65	0.6769	2.22281
Ukraine	103	1.4563	5.08897

The following table (Table 5) reports effect size measures (*d*) for all statistically significant (*p* < 0.05) pairwise country mean comparisons by Tukey test for the SiteUse data. Effect sizes were calculated as the mean difference divided by the weighted standard deviation across all groups. The rows and columns are ordered from lowest to highest mean scores. Effect sizes ranged from large to negligible. These results showed that we had enough statistical power to detect effects accounting for less than one percent of the variance (a small effect according to Cohen’s guidance), assuming adequate sample sizes. Ukraine, Slovakia, Turkey, Romania, and Serbia all showed significantly higher rates for using outsourcing sites than other countries.

**Table 5:** Standardized Mean Differences for Significant Differences in Mean SiteUse Scores by Country*

	Republic of Serbia	Romania	Turkey	Slovakia	Ukraine
Sweden	0.20	0.33	0.44	0.51	1.12
United Kingdom					1.11
Australia	0.19	0.31	0.42	0.50	1.11
Italy					1.04
Chile		0.23	0.34		1.02
Czech Republic			0.34		1.02
Hungary					1.01
New Zealand					1.00
United States					0.96
Republic of Serbia					0.92
Romania					0.79
Montenegro					0.71
Turkey					0.68
Slovakia					0.61

Ukraine had the largest mean score by a wide margin. Most of the effect sizes involving Ukraine were large (> 0.80). The mean for Ukraine was also significantly greater than the means for 14 of the other countries. By contrast, Sweden and Australia had the lowest means, significantly lower than the means of five other countries. All of the effect sizes that did not involve Ukraine were within the range of small effects, or less.

Further factors considered for correlations, in response to R1, were the enrolled discipline/s being studied, language of study and gender. For Discipline, the one-way ANOVA test was statistically significant (*p* < 0.0005) for SiteUse. Table 6 reports means and standard deviations (SD) of the SiteUse scores for each discipline.

**Table 6:** Means and Standard Deviations of SiteUse Scores by Discipline

Discipline	Mean	SD
Health	0.0991	0.83826
Languages	0.1183	0.69712
Engineering	0.1347	0.65247
Architecture	0.1357	0.57790
Education	0.1448	0.68783
Creative Arts	0.1640	1.20953
Society/Culture	0.1677	1.05622
Sciences	0.1686	1.13707
Law/Justice	0.2053	1.37332
Economics	0.2520	1.72887
Info Tech	0.3364	1.60352
Management/Commerce	0.4029	2.75442
Agriculture	0.6080	4.43573
Hospitality/Personal	1.0792	5.48395

Post hoc Tukey tests determined that the mean for Hospitality and Personal Services was the highest and significantly greater than the mean for every other category except Agriculture, demonstrating that students enrolled in these disciplines were more likely to engage in formal outsourcing than other disciplines. The mean for Health was the lowest and significantly less than the means for Agriculture and Management/Commerce, as well as Hospitality and Personal Services. No other significant mean differences were uncovered through post hoc testing. The effect size measures for these significant differences are reported in the following data (Table 7). Most of the effects for Hospitality and Personal Services were in the medium range (0.50—0.80). The remaining effect sizes were in the small range (0.20-0.50) or less.

**Table 7:** Standardized Mean Differences for Significant Differences in Mean SiteUse Scores by Discipline

	**Hospitality/Personal**	**Health**
Health	0.62	
Languages	0.60	
Engineering	0.59	
Architecture	0.59	
Education	0.59	
Creative Arts	0.57	
Society/Culture	0.57	
Sciences	0.57	
Law/Justice	0.55	
Economics	0.52	
Info Tech	0.47	
Management/Commerce	0.42	0.19
Agriculture		0.32

For Gender, the one-way ANOVA test was statistically significant (*p* = 0.019). Males reported significantly higher rates of use of these sources than females. However, the difference did not reach the level of Cohen’s recommendation for a small effect size (*d* = 0.07). For Language, the one-way ANOVA test was not statistically significant (Welch *p* = 0.176) with the mean for second language students (0.3522) higher than the mean for first language students (0.2408). The effect size was minimal (*d* = 0.06).

### Regression

The above analyses all address the relationships between one predictor and our outcome variable. However, these analyses do not account for covariance between predictors. For example, if there are differences across disciplines in the use of these sites, it is plausible that there are differences across disciplines in how often participants believe other students use these sites. For this reason, we ran a regression using all the predictors of at least one percent of the outcome variable.

**Table 8:** Test of Between Subjects Effects for SiteUse

Source		Type III Sum of Squares	df	Mean Square	F	Sig	Partial Eta Squared
Intercept	Hypothesis	92.343	1	92.343	52.775	0.000	0.040
	Error	2224.558	1271.350	1.750^a^			
Country	Hypothesis	164.536	14	11.753	8.443	0.000	0.017
	Error	9250.817	6646	1.392^b^			
Discipline	Hypothesis	32.279	13	2.483	1.784	0.040	0.003
	Error	9250.817	6646	1.392^b^			
OthersSiteUse	Hypothesis	22.336	1	22.336	16.046	0.000	0.002
	Error	9250.817	6646	1.392^b^			
UseRate	Hypothesis	910.172	77	11.820	8.492	0.000	0.090
	Error	9250.817	6646	1.392^b^			

^a^0.034 MS(Rate) + 0.966 MS(Error)

^b^MS(Error)

One continuous variable (UseRate), and three categorical variables (OthersSiteUse, Country, and Discipline) each individually explained more than one percent of the variance in participants’ engagement in formal outsourcing in the bivariate analyses. These four predictors were entered into a multiple regression using the UNIANOVA command in SPSS (see Table 8). This command provides regression analysis results when only one outcome variable is involved with multiple predictor variables. The three categorical predictors were entered into the analysis as fixed factor variables, while the one continuous predictor was entered as a covariate. For categorical predictors, the category with the highest mean response was used as the reference category if there were more than two categories in the variable.

The main effects model for this analysis yielded statistically significant results for all four of the predictor variables (*p* < 0.05). However, only Country (1.7%) and UseRate (9.0%) accounted for more than one percent of variance. In other words, the extent to which participants believed other students were engaging in these practices was the strongest predictor of their own use of essay mills, while differences across countries were the second strongest predictor.

## Discussion

Answering our first research question: ‘Are there personal or situational variables that have a correlation with increased usage of assignment outsourcing sites?’, we discovered that most categorial variable predictors (knowledge of others’ use of sites, country of study, and discipline), were significantly related to formal outsourcing. For categorical variables, several differences in site use across countries yielded large effect sizes, accounting for 16%, or more of the variance in formal outsourcing. However, among continuous predictors, only the rate that participants believed other students were using sites accounted for at least 1% of the variance in site use, or a small effect size.

We found that there were different patterns in usage of sites internationally, with large differences seen between some countries (Sweden and Ukraine had the largest). Small to medium effect sizes for differences in site use across disciplines were found; country of study was related to level of engagement with outsourcing. Students in Ukraine had far higher mean usage of sites compared to other countries. Prior studies analysing Ukrainian student perceptions of academic dishonesty and cheating behaviours, found Ukrainian students reported more cheating than students in other countries (Chudzicka-Czupała et al., 2013; Stephens et al., 2010).

Whilst three disciplines (Hospitality and Personal Services, Agriculture and Management/Commerce) were found to be statistically significant in predicting student engagement with outsourcing sites, only Hospitality and Personal Services had a medium effect size. These results are consistent with prior research which found more cheating in marketing, business and administration courses (Chapman et al., 2004; Selwyn, 2008, respectively). McCabe et al., (2006) reported that 56% of business students admitted to some form of cheating compared to 47% of non-business students. However, contrary to this Klein et al., (2007) did not find any significant difference between overall self-reported cheating rates of business students and other professional students. Given the variance found in different studies, a future research study could undertake meta-analysis of all research considering relationships between discipline and engagement with contract cheating.

Our data found that demographic variables were significantly related to site use but did not have substantial effect sizes. For example, whilst we found that males outsourced from sites significantly more than females, this did not have a large enough effect size to be considered a predictor variable. This is contrary to some research which has found gender (male) to be a predictor of cheating behaviours (Genereux & McLeod, 1995; McCabe & Trevino, 1997). However, other research has found that gender does not have an effect of cheating behaviours (Franklyn-Stokes & Newstead, 1995). Similarly, we found higher rates of site usage amongst second language learners compared to first language learners. However, these results did not find that second language learning was a predictor of outsourcing as effect sizes were minimal. Conversely, Bretag et al., (2018a, b) found strong correlations between students studying in their second language and cheating rates (see also Rigby et al., 2015).

As a result of the outcomes from the testing for all situational and personal variables, we were able to positively confirm our second research question: ‘Do students who outsource through formal methods have higher rates of known or perceived cheating amongst their peer groups and the general student population?’. To test the relative strengths of the predictor variables, we included all of the individual predictors that accounted for at least one percent of the variance in site use into a regression model. In this combined model, all of the predictor variables were statistically significant. How often participants believed that the general student population were using essay mills predicted 9% of the variance in use of these sites. Differences between countries accounted for another 1.7% of that variance. Two other predictors failed to account for 1% or more of the remaining variance. Prior research demonstrated the correlation between self-reported cheating rates and knowledge of others cheating (Ives & Giukin, 2020; Ives et al., 2017; O’Rourke et al., 2010; Rettinger & Kramer, 2009; Teodorescu & Andrei, 2009).

Universities can take some practical action from these results. In considering our data, perhaps if students think higher rates of students are cheating, a normalisation of cheating is created. Despite recent legislative interventions, Awdry et al., (2021) also found that students were not deterred by potential legal action and thought that students were outsourcing irrespective of the legality of the act. Given the promotion of these formal outsourcing sites as normal study aids (Harrison et al., 2020; Medway et al., 2018; Rowland et al., 2018), universities must promote education and conversations around ethics and integrity as an integral part of their culture. Integrity and ethics being integral to university experience has been endorsed by many (Gullifer & Tyson, 2010; McCabe & Pavela, 2004; Morris, 2016, 2018; Morris & Carroll, 2016; Rogerson, 2017), and should be strengthened and combined with messaging about the pitfalls of using these sites, which may also help to alter students perceptions about how widespread their use is.

Intervening in the false perceptions of the quantity of students cheating in this manner is one area which can be easily addressed through the publication of institutional data on detection rates and open conversations among the student body with those who have, have not and who had thought about cheating. Highlighting the importance of conversations around honesty, McGloin and Thomas (2016) found that positive social behaviours were based more on first hand/witnessed experiences, rather than deviant behaviours which may be based on assumptions or second-hand information. Encouraging open conversations with students to state why they would not cheat would be one step towards promoting positive social behaviours.

As we found in our data, the acceptable reasons to cheat did not represent a strong predictor for formal outsourcing, although they were statistically significant. Therefore, we find a negative response to our final research questions: ‘Do the students who outsource their assignments from sites agree with more factors as to why it is acceptable to cheat?’. This points to a need to focus on the peer-related factors. Given the inflated perception of student’s beliefs of others’ cheating, it may well be worth discussing institutional detections rates with students, and what outcomes/sanctions may be applied. Not only will this help reduce potentially inaccurate perceptions of rates of engagement in formal outsourcing but will demonstrate that the university does detect outsourced assignments. As Rigby et al. (2015) found, when confronted with the highest-level sanctions, students never opted to pay for an assignment. A common factor in students seeking unauthorised help may often be perceived as lack of staff action and likelihood of being caught (Akbulut et al., 2008; Szabo & Underwood, 2004).

Whilst predictors in themselves do not offer practical solutions, they allow educators to consider all elements related to cheating; this knowledge is something which many interventions to-date have not had available, nor used, and may aid in design strategies to reduce student’s engagement in formal outsourcing by targeting these correlational factors. As noted by (Ives & Nehrkorn, 2019), research on interventions has shown some positive outcome in relation to awareness of academic integrity, but many did not assess improvement in behaviours. Further, studies did not test the actual effectiveness of the range of approaches taken. Only at the introduction and trial of interventions are we able to assess their effectiveness in reducing contract cheating and assignment outsourcing.

## Limitations

Due to the snowballing of the survey through convenience sampling methods we were unable to ensure a representative sample or large response rate from some countries. Whilst we were able to test for significance in the above analysis, it would provide further strength to test with a more representative research population. Due to the different response rates from countries, we determined that the most appropriate method was to combine all countries together for analysis of other demographic and situational factors. We acknowledge that statistical significance may not provide practical solutions for educators to enable them to apply outcomes to their local context. We have considered these practical limitations against our analysis in the discussion and recommendations, and readers should assess the practicality of the results in considering their strategies to reduce academic misconduct of this nature.

For purposes of analysis we combined all responses to the questions asking about site usage, into one category, however, for future analysis it may be useful to separate each type of site outsourcing to determine whether some are more affected by the predictor variables than others. It would be interesting to explore this for the peer-sharing sites. We recommend that for those countries who did not release the survey widely, that a collective release of the survey in multiple universities is promoted as a national research project, to enable more specific analyses, and country-relevant responses. This may be particularly pertinent due to the survey data having been collected prior to the COVID-19 pandemic and learning environments having changed dramatically since the responses were gathered. It may also be worthwhile to undertake a specific comparison of practices between those countries with the lowest and highest outsourcing rates to determine if there are any educational/institutional practices or structures which may encourage or deter cheating.

## Conclusion

Our research has demonstrated that whilst there were differences found between countries, with some showing a higher propensity to engage in cheating through formal outsourcing, the variable most strongly able to predict engagement in this behaviour was the rate at which respondent’s believed other students were cheating (irrespective of whether the rate is realistic or not). This belief that others are cheating must be accounted for by educators who are designing strategies to reduce students’ use of these sites. Engagement in ethics and promotion of positive conversations around acceptable sources of help are just one way to start. However, any effective strategy will need a whole of institution approach. Ensuring faculty staff have the education, training and tools to detect suspected cases of outsourcing and present appropriate evidence for decision-makers will help increase detection rates. Publishing institutional data on the numbers of students caught may help to mitigate some of the inaccurate beliefs around systemic cheating some students may have, and which can ultimately lead to them outsourcing. Understanding the correlates of contract cheating is the first step towards intervening to divert their effects towards honest behaviours.

## Data Availability

The complete data set is not publicly available.

## Appendix 1

Means and Standard Deviations of SiteUse Scores by Country.

**Table:**

Country	N	Mean	SD
Bulgaria	17	0.0000	0.00000
Sweden	1142	0.0193	0.26075
United Kingdom	63	0.0317	0.25198
Australia	1340	0.0358	0.33052
Germany	30	0.0667	0.25371
Malaysia	12	0.0833	0.28868
Italy	237	0.1266	0.82391
Singapore	21	0.1429	0.65465
Czech Republic	575	0.1461	0.69056
Chile	1048	0.1498	0.62538
Hungary	208	0.1538	0.91966
New Zealand	76	0.1711	1.17062
Croatia	29	0.2069	0.67503
United States	99	0.2424	0.90453
Republic of Serbia	1211	0.2733	1.85609
Romania	359	0.4401	1.35826
Montenegro	64	0.5469	1.79885
Turkey	166	0.5783	2.28365
Slovakia	65	0.6769	2.22281
United Arab Emirates	35	1.0000	4.61455
Ukraine	103	1.4563	5.08897

## Appendix 2

Standardized Mean Differences for Significant Differences in Mean SiteUse Scores by Country*

**Table:**

	Republic of Serbia	Romania	Turkey	Slovakia	Ukraine	United Arab Emirates
Sweden	0.20	0.33	0.43	0.49	1.12	1.13
United Kingdom					1.11	1.12
Australia	0.17	0.31	0.42	0.47	1.11	1.12
Germany					1.10	1.12
Malaysia						
Italy					1.03	1.05
Chile		0.23	0.33		1.02	1.03
Singapore					1.02	
Czech Republic			0.33		1.01	1.03
Hungary					1.01	1.03
New Zealand					1.00	1.02
Croatia					0.99	
United States					0.94	0.96
Republic of Serbia					0.92	0.93
Romania					0.79	
Montenegro					0.70	
Turkey					0.69	
Slovakia					0.63	
Ukraine						
